# Prognostic and predictive value of Ki-67 in triple-negative breast cancer

**DOI:** 10.18632/oncotarget.9075

**Published:** 2016-04-28

**Authors:** Wei Wang, Jiayi Wu, Peifeng Zhang, Xiaochun Fei, Yu Zong, Xiaosong Chen, Ou Huang, Jian-Rong He, Weiguo Chen, Yafen Li, Kunwei Shen, Li Zhu

**Affiliations:** ^1^ Comprehensive Breast Health Center, Ruijin Hospital, Shanghai Jiaotong University School of Medicine, Shanghai 200025, P.R. China; ^2^ Department of Information Engineering, Shanghai Jiaotong University, Shanghai, 200240, P.R. China; ^3^ Pathology Department, Ruijin Hospital, Shanghai Jiaotong University School of Medicine, Shanghai 200025, P.R. China

**Keywords:** breast carcinoma, hormonal receptor negativity, human epithelial growth factor receptor 2 negativity, proliferation index, platinum

## Abstract

This study was to investigate the prognostic role of Ki-67 in further classification of triple negative breast cancer (TNBC), and to test whether high expression level of Ki67 can predict benefit from carboplatin. From January 2004 to December 2012, 363 patients operated for TNBC were identified through the institutional clinical database. After a median follow-up time of 34 months (5.2–120.0 months), 62 patients (17.1%) had relapses and 33 patients (9.1%) died of breast cancer. In univariate analysis, high Ki-67 index as well as larger tumor size and lymph node involvement was associated with shorter disease-free survival (DFS) and overall survival (OS). In multivariate analysis, high Ki-67 is an independent risk factor for DFS (Risk Ratio, RR: 2.835, 95% confidence interval, 95% CI: 1.586–5.068, *P* < 0.001) and OS (RR: 3.180, 95% CI: 1.488–6.793, *P* = 0.003). When analyzing the 3-year DFS by Ki-67 distribution, Subpopulation Treatment Effect Pattern Plot analysis showed a beneficial effect of carboplatin in patients with high Ki-67 index. In conclusion, TNBC is probably a heterogeneous disease with different characteristics and prognosis, and may be further subdivided according to the Ki-67 expression levels. Patients in the high Ki- 67 group seem to benefit more from treatment with carboplatin, but this needs to be further verified.

## INTRODUCTION

Triple negative breast cancer (TNBC) is a subgroup of breast cancer lacking estrogen receptor (ER) and progesterone receptor (PR) expression as well as human epithelial growth factor receptor 2 (HER2) amplification. From the histological perspective, TNBC is a common immunohistochemical (IHC) status for a number of tumors with heterogeneous clinical presentations [1]. Recent study identified six TNBC subtypes that display unique profiles [2]. Given the biological diversity within TNBC, it is essential to identify subtypes with a better prognosis which may be spared intensive adjuvant therapy, and those in greatest need of more aggressive regiments.

Tumor proliferative activity, an important cellular function, is closely related to tumor behavior in breast cancer [3]. Various techniques have been developed to assess the proliferation rates, including mitotic count, estimation of the cell fraction in S-phase of cell cycle and IHC determination of proliferation-associated antigens. Ki-67 is one of the most widely used IHC proliferation antigen and has been confirmed as an independent predictive and prognostic factor in early breast cancer [4, 5]. The value of Ki-67 is an important parameter in sub-classifying luminal tumors into a good prognosis luminal A subgroup and a poor prognosis luminal B subgroup according to St Gallen International Expert Consensus [6]. While the prognostic value of the Ki-67 level in TNBC is yet unclear.

Treatment for TNBC has been challenging. TNBC is generally considered to exhibit a more aggressive clinical behavior and higher risk of tumor relapse and mortality [7, 8] compared with its nontriplenegative counterparts. Besides, the absence of well-defined molecular targets makes it worse and the only treatment strategy is cytotoxic agents. Platinum salt is one of the emerging agents in the treatment of TNBC. Many investigators have explored the role of cisplatin and carboplatin for the treatment of TNBCs in neo-adjuvant and metastatic setting [9–14], while the addition of carboplatin in early stage disease still lacks strong evidence. One important question is whether all TNBC patients need to be exposed to carboplatin, with its toxic effects and high rate of treatment discontinuation; or whether all patients with TNBC would benefit similarly from a platinum salt [2, 15].

The aim of this study was to investigate the role of Ki-67 in further classification of TNBC into subtypes with different prognosis, and whether the expression level of Ki-67 can predict benefit of TNBC from carboplatin in adjuvant setting.

## RESULTS

A total of 363 TNBC patients were included in this study. Median age was 55 years (range 23–86). Two hundred and seventy five patients (75.8%) underwent mastectomy; 324 (89.3%) patients received chemotherapy. Chemotherapy regimens included EC (Epirubicin 100 mg/m^2^ IV day 1, Cyclophosphamide 600 mg/m^2^ IV day 1, cycled every 21 days for 4 cycles), EC-T (Epirubicin 100 mg/m^2^ IV day 1, Cyclophosphamide 600 mg/m^2^ IV day 1, cycled every 21 days for 4 cycles followed by Docetaxel 100 mg/m^2^ IV on day 1, cycled every 21 days for 4 cycles), TEC (Docetaxel 75 mg/m^2^ IV day 1, Epirubicin 75 mg/m^2^ IV day 1, Cyclophosphamide 600 mg/m^2^ IV day 1, cycled every 21 days for 6 cycles), TC (Docetaxel 75 mg/m^2^ IV day 1, Cyclophosphamide 600 mg/m^2^ IV day 1, cycled every 21 days for 4 cycles), EC-wPCb (Epirubicin 100 mg/m^2^ IV day 1, Cyclophosphamide 600 mg/m^2^ IV day 1, cycled every 21 days for 4 cycles followed by Paclitaxel 75 mg/m^2^ IV day 1, Carboplatin 2·0 area under curve, cycled every week for 12 cycles) and wPCb (Paclitaxel 75 mg/m^2^ IV day 1, Carboplatin 2·0 area under curve, cycled every week for 12 cycles). Regimens containing both anthracycline and taxanes were used in 177 patients (48.8%) and 58 (16.0%) patients received platinum-containing regimen. One hundred and seventy one patients (47.1%) received radiotherapy.

Out of 363 triple negative tumors, 317 (87.3%) were histologically identified as invasive ductal carcinomas, 17 (4.7%) as apocrine carcinomas, 9 (2.5%) as medullary carcinomas, 7 (1.9%) as metaplastic carcinomas, 2 (0.6%) as invasive lobular carcinomas, 3 (0.8%) as neuroendocrine carcinoma, 3 (0.8%) as invasive papillary carcinoma, 2 (0.6%) as adenocystic carcinoma, 1 (0.3%) as myoepithelial carcinoma, 1 (0.3%) as malignant phyllodes tumor and 1 (0.3%) as mucinous carcinoma.

The median Ki-67 expression level was 40%. With 40% as the cutoff value of Ki-67 index, 196 patients (54.0%) were classified as Ki-67 low expression and 167 patients (46.0%) as high expression. Patients' characteristics in association with Ki-67 expression level are described in Table 1. High expression level of Ki- 67 in TNBC was more common in IDC compared with non-IDC (*p* < 0.001) and was associated with younger age (*p* < 0.001) and higher tumor grade (*p* < 0.001). The correlation between tumor size (*p* = 0.177), lymph node metastasis (*p* = 0.136) and Ki-67 expression level is not significant. More patients in Ki-67 high-expression subgroup received chemotherapy (*p* = 0.007).

**Table 1:** Correlation of Ki-67 and patient characteristics

Characteristics	Low Ki-67 N (%)	High Ki-67 N (%)	*P*-value
Age			0.000
≤ 55	83 (42.3%)	106 (63.5%)	
> 55	113 (57.7%)	61 (36.5%)	
Histology			0.000
IDC	159 (81.1%)	158 (94.6%)	
Non-IDC	37 (18.9%)	9 (5.4%)	
Tumor size (cm)			0.177
T1	98 (50.0%)	73 (43.7%)	
T2	78(39.8%)	82 (49.1%)	
T3–4	20 (10.2%)	12 (7.2%)	
ALN status			0.136
N0	138 (70.4%)	105 (62.9%)	
N1	21 (10.7%)	30 (18.0%)	
N2	28 (14.3%)	20 (12.0%)	
N3	9 (4.6%)	12 (7.2)	
Tumor Grade			0.000
I	27 (13.8%)	3 (1.8%)	
II	116 (59.2%)	48 (28.7%)	
III	53 (27.0%)	116 (69.5%)	
Breast surgery			0.175
Mastectomy	154(78.6%)	121 (72.5%)	
Lumpectomy	42 (21.4%)	46 (27.5%)	
Chemotherapy			0.007
Yes	167 (85.2%)	157 (94.0%)	
No	29 (14.8%)	10 (6.0%)	
Radiotherapy			0.361
Yes	88 (44.9%)	83 (49.7%)	
No	108 (55.1%)	84 (50.3%)	

**Figure 1:**
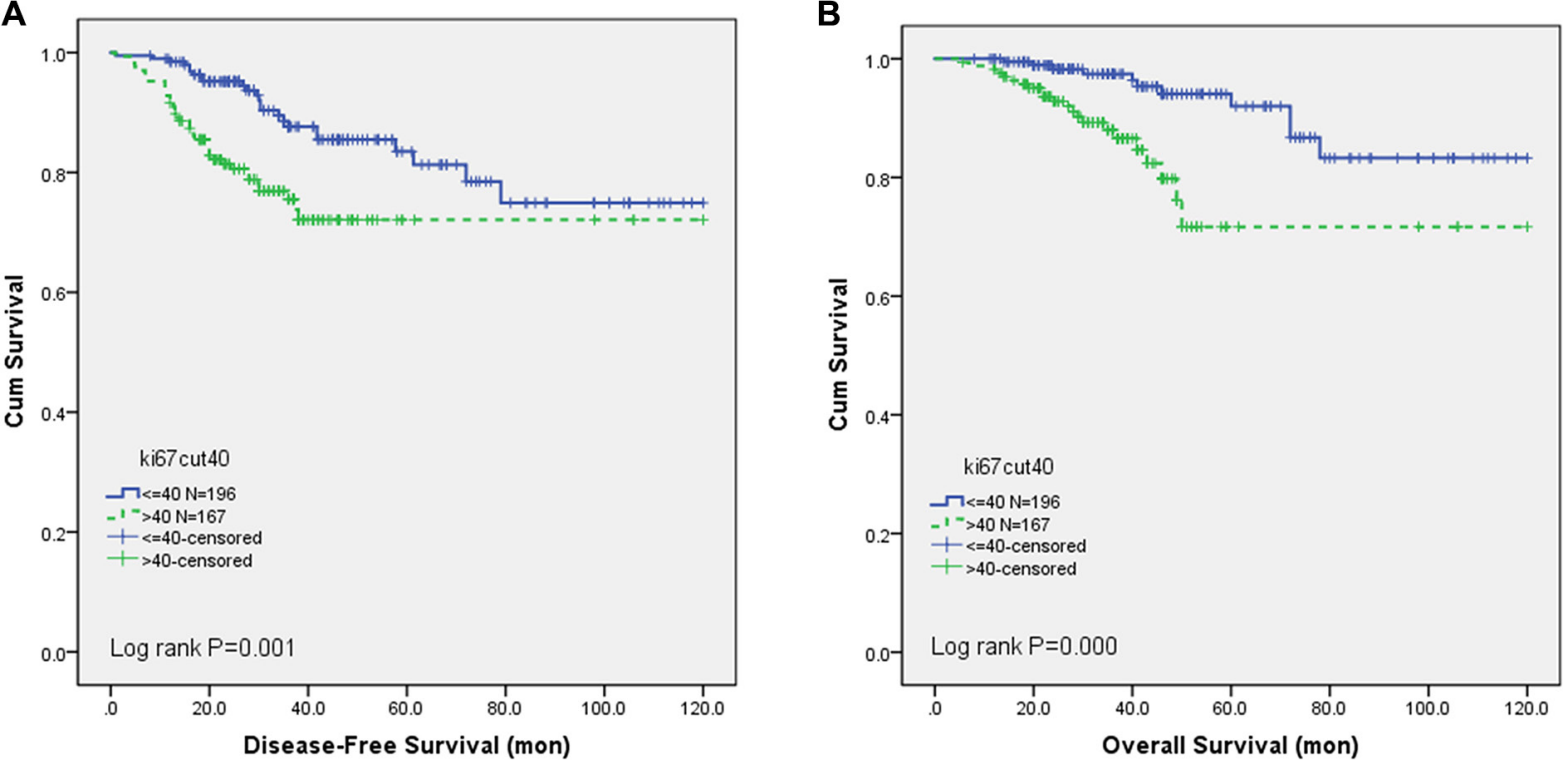
Disease-free survival and overall survival by Ki-67 expression level (**A**) The 3-year DFS was significantly better in low Ki-67 group than in the high Ki-67 group (90.8% vs 78.4% Log-rank *p* = 0.001) and (**B**) A poorer 3-year OS was also detected in high Ki-67 group (98.0% vs 90.4% Log-rank *p* = 0.000).

After a median follow-up time of 34.0 months (5.2– 120.0 months), 62 first events were observed (17.1%), with 24 (12.2%) in Ki-67 low-expression level group and 38 (22.8%) in high-expression level group (X^2^ = 11.372, *p* = 0.001). The 62 first events included 53 relapses with 30 locoregional recurrences and 44 distant metastases and 9 other events with 6 contralateral breast cancers. All the locoregional recurrence lesions and contralateral breast lesions were proved by using either fine needle aspiration or core needle biopsy. Thirty-three patients (9.1%) died during the follow-up time and Ki-67 high expression patients had a higher rate of death (13.2% vs 5.6%, *X*^2^=13.368, *p* < 0.001). Meanwhile, high Ki- 67 index was significantly associated with poorer 3-year DFS (90.8% vs 78.4% Log-rank *p* = 0.001; Figure 1A) and OS (98.0% vs 90.4% Log-rank *p* = 0.000; Kaplan-Meier Curve Figure 1B).

In univariate analysis, only high Ki-67 expression, larger tumor size, lymph node positivity were associated with shorter DFS and OS, while other clinical pathological characteristics, such as age, histological subtype and tumor grade did not influence the prognosis. In the multivariate analysis, Ki-67 is an independent prognostic factor for DFS (Risk Ratio, RR: 2.835, 95% confidence interval, 95% CI: 1.586–5.068, *P* < 0.001) and OS (RR: 3.180, 95% CI: 1.488–6.793, *P* = 0.003). Results from the univariate analysis and final multivariate Cox regression model are presented in Table 2A, 2B.

**Table 2A:** Multivariate cox regression of DFS

	Univariate		Multivariate	
	HR (95% CI)	*P* value	HR (95% CI)	*P* value
Tumor size		0.026		0.030
T1	1.000		1.000	
T2	2.292 (1.234, 4.257)	0.009	2.226 (1.206, 4.111)	0.011
T3–4	2.321 (0.972, 5.543)	0.058	2.299 (0.972, 5.437)	0.058
ALN status		0.000		0.000
N0	1.000		1.000	
N1	2.120 (1.046, 4.298)	0.037	2.084 (1.044, 4.158)	0.037
N2	1.549 (0.657, 3.655)	0.317	1.628 (0.765, 3.463)	0.206
N3	7.332 (2.994, 17.960)	0.000	7.834 (3.589, 17.099)	0.000
Grade		0.102		0.089
I	1.000		1.000	
II	2.302 (0.850, 6.335)	0.101	2.378 (0.873, 6.477)	0.090
III	1.817 (0.996, 3.313)	0.052	1.847 (1.015, 3.363)	0.045
Ki-67		0.000		0.000
low	1.000		1.000	
high	2.952 (1.623, 5.274)	0.000	2.835 (1.586, 5.068)	0.000

**Table 2B:** Multivariate cox regression of OS

	Univariate		Multivariate	
	HR (95% CI)	*P* value	HR (95% CI)	*P* value
Tumor size (cm)		0.009		0.008
T1	1.000		1.000	
T2	4.787 (1.753, 13.074)	0.002	4.855 (1.797, 13.117)	0.002
T3–4	3.508 (0.921, 13.355)	0.066	3.739 (1.081, 12.935)	0.037
ALN status		0.000		0.000
N0	1.000		1.000	
N1	2.663 (0.998, 7.105)	0.050	2.611 (1.021, 6.675)	0.045
N2	1.734 (0.507, 5.933)	0.380	1.403 (0.484, 4.073)	0.533
N3	13.878 (4.071, 47.310)	0.000	11.104 (4.119, 29.935)	0.000
Ki-67		0.004		0.003
low	1.000		1.000	
high	3.558 (1.488, 8.507)	0.004	3.180 (1.488, 6.793)	0.003

**Figure 2:**
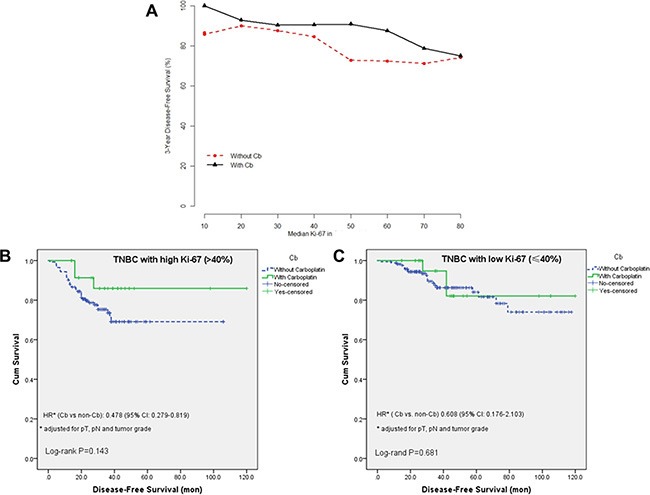
The effect of carboplatin treatment on DFS (**A**) Subpopulation Treatment Effect Pattern Plot (STEPP) of 3-year Disease-free survival. (**B**) Disease-free survival in the high Ki-67 (Ki-67 > 40%) group according to carboplatin treatment. (**C**) Disease-free survival in the low Ki-67 (Ki-67 ≤ 40%) group according to carboplatin treatment. Univariate log-rank test *p*-values and Hazard Ratios (HR) (carboplatin vs non carboplatin) were reported.

When analyzing the 3-year DFS by Ki-67 distribution, STEPP analysis showed a possible beneficial effect of Carboplatin in patients with highly proliferative tumor (Ki-67 > 40%) (Figure 2A). Figure 2B and Figure 2C present the observed DFS proportion respectively for patients with “high” and “low” Ki-67, stratified by treatment group. In patients with low Ki-67 breast cancer, the use of carboplatin adds little, if any, benefit to the 3-year DFS (HR: 0.608, 95% CI: 0.176–2.103). However, patients in the high Ki-67 group seems have a remarkable better 3-year DFS rates when treated with carboplatin (HR: 0.478, 95% CI: 0.279–0.819). The interaction between Ki-67 and treatment was not statistically significant (*p* = 0.346).

## DISCUSSION

TNBC is a group of tumors with poor prognosis because of aggressive tumor biology and lack of targeted agents [16]. Better understanding of its biological behavior is essential to improve the outcomes for TNBC patients. In this study, we retrospectively reviewed 363 patients to analyze the correlation between Ki-67 expression level with clinicopathological characteristics and prognosis of TNBC. All the patients coming from one center ensured that the test quality of pathological biomarkers and treatment decision are basically stable.

The use of Ki-67 as a prognostic marker in breast cancer has been widely investigated, but only a few studies have investigated it in the triple negative subgroup [17–19]. Some researchers [18] explored the prognostic value of Ki- 67 in the whole cohort of breast cancer, but the number of cases in TNBC and Her2+ classes was quite small and this may limit the ability of Ki-67 to identify clinically distinct subclasses. A Korean group [19] study showed that in preoperative setting, a high Ki-67 expression (≥ 10 %) was significantly associated with poor relapse-free survival and overall survival in TNBC in spite of a higher pathologic complete response (pCR) rate. Munzone et al. [20] reported that Ki-67 labeling index was associated with different prognosis subgroups in node-negative TNBC with a cut-off value of 35%. In line with these results, our study found that high expression of Ki-67 (> 40%) is significantly correlated with a worse prognosis in TNBC patients, irrespective of the tumor size and lymph node status.

Ki-67 measurement by IHC is a low cost method suitable for widespread use in clinical practice [21]. International Ki-67 in Breast Cancer Working Group has proposed guidelines for the analysis, reporting, and use of this potentially important marker based on current evidence [22]. The guidelines were strictly followed in this study, which guaranteed the value of it. In another retrospective study from our center [23], high expression of Ki-67 was correlated with early recurrence in Luminal B/Her2 negative breast cancer, with a cut-off value of 30%. This may reflect the stability and reliability of the test of Ki-67 in one center.

Cut-off points of Ki-67 index employed in clinical trials and studies differed widely [17–18, 20–21], ranged from 10% to 61%. Since baseline Ki-67 values for triple negative and HER2 positive tumors are much higher than for luminal tumors [18], cut-offs selection of Ki-67 might be more apparent if it was considered within each subgroups respectively. We selected the median of Ki- 67 as the cut-off value in this study, which was widely adopted in other researches [24]. In view of the inter-observer and across-labs variability, much more evidence is needed to set an appropriate cut-off point of Ki-67 for TNBC.

The follow-up time of our study is relatively short. However, despite of a median 34-month follow-up, Ki-67 expression level shows its independent prognostic value in TNBC. This might be attributed to the early recurrence pattern of TNBC within the first three years of follow-up [25, 26]. In this study, 94.3% (50/53) relapse occurred in the first three years following the surgery.

A key point for usage of platinum-contained regimen would be the selection of the right patient. There is a well-described association between TNBCs and BRCA germline mutations [27]. Neoadjuvant trials have shown high rates of pCR among BRCA1-associated breast cancers treated with cisplatin [28]. However, the routine clinical application of BRCA gene tests still has some difficulties. While the test of Ki-67 is more convenient and economical and might be a good alternative.

In GeparSixto clinical trial [10], the addition of neoadjuvant carboplatin to a taxane-anthracycline regimen significantly increases the pCR proportion of TNBC patients. Subgroup analysis showed that Odds radio favor carboplatin in high Ki-67 group (> 20%) is 1.40 (95%CI: 0.968–2.02), higher than in the low Ki-67 group (OR: 1.09, 95% CI: 0.490–2.4). Similarly, our study showed a possible beneficial effect of carboplatin in patients with highly proliferative tumor (Ki-67 > 40%) in the adjuvant setting. But this trend still needs to be verified in further prospective, well-balanced studies with large sample size.

One possible limitation of this study could be related to the heterogeneity of the adjuvant treatment, as not all patients received the same regimen. However, we can assess that among the patients who received chemotherapy, the majority (72.5%) received an anthracycline-containing regimen and more than half of them (55.6%) received regimens containing both anthracycline and taxanes.

In conclusion, TNBC seems to be a heterogeneous group with different clinical outcomes. TNBC with high proliferation potential should be followed-up more frequently within three years, and might be a candidate for additional postoperative treatments with different mechanisms, such as carboplatin.

## MATERIALS AND METHODS

### Patients

We collected information on consecutive breast cancer patients undergoing breast surgery between January 2004 and December 2012 in Shanghai Ruijin Hospital through the breast cancer database at the Comprehensive Breast Health Center. The protocol was approved by the Ethical Committees of Shanghai Ruijin Hospital and all the patients provided their written informed consents to participant this study before the clinical and pathological data were collected.

A total of 363 TNBC patients were retrospectively investigated. The baseline data including age, tumor characteristics (tumor size, lymph node metastasis, distant metastasis, tumor grade, pathological stage, ER/PR/HER2 expression and histological type) and surgical information were retrieved. Treatment decision for every patient was made by daily multidisciplinary meeting that was attended by surgeons, medical oncologists, radiation oncologists, and pathologists.

### Pathology methods

Tumors were classified histologically according to the World Health Organization Classification of Tumors [29]. Histological grade was evaluated according to Elston and Ellis scoring system [30]. IHC staining of ER, PR, HER2 and Ki-67 was routinely carried out by using Ventana BenchMark XT system (Ventana Medical Systems, Tucson, AZ). IHC staining was performed on 4-μm slices of formalin-fixed paraffin-embedded (FFPE) tissue sections with primary antibodies against ER (SP1, 1:100, Dako, Denmark), PR (PgR 636, 1:100, Dako, Denmark), HER2 (4B5, Roche, Switzerland), Ki- 67(MIB- 1, 1:100, Dako, Denmark). IHC expression of HER2 was scored as follows: 0 (no staining or faint membrane staining), 1+ (faint membrane staining in >10 % of tumor cells, incomplete membrane staining), 2+ (weak to moderate membrane staining in > 10% of tumor cells), and 3+ (uniform, intense membrane staining of > 30% of invasive tumor cells). A Fluorescence *in situ* hybridization (FISH) test for HER2 gene amplification was routinely ordered when HER2 was IHC 2+. FISH was performed using the PathVysion HER-2 DNA FISH Kit (Vysis Inc, Downers Grove, IL) according to the manufacturer's instructions.

All histologic and IHC tumor slides were evaluated by two pathologists. Histological grades and all biological features were evaluated based on the invasive components.

The cutoff for ER positivity and PR positivity was 1% positive tumor cells with nuclear staining [31]. Positive for HER2 was either IHC HER2 3+ or FISH amplified (ratio of HER2 to CEP17 of ≥ 2.0 or average HER2 copy number ≥ 6.0 signals/cell) [32]. The Ki-67 index was expressed as the percentage of positively nuclear staining cells among at least 1000 invasive cells in the area scored. Staining intensity was not relevant [22].

### Follow-up and statistical analysis

Breast cancer relapse was defined as the first proven invasive local/contralateral breast, regional, or distant recurrence in any site [33]. The disease-free survival (DFS) was defined as the interval from the date of the primary surgery to the first relapse, second primary non-breast invasive cancer or death attributable to any cause. Overall survival (OS) was defined as the time from the date of primary surgery to the time of death regardless of breast cancer related or not.

All *p* values less than 0.05 were considered to indicate statistical significance. All statistical tests were two-sided, with the confidence interval of 95%. Chi-Square test was employed for categorized variables (Fisher's exact test when the Chi-square test was unavailable). Survival curves were plotted by Kaplan-Meier method. Log-rank test was used to determine the associations between individual variables and survival, logistic regression modeling to examine the association of tumor features with Ki-67 expression level and Cox proportional hazards regression analyses to identify significant prognostic factors in TNBC. Statistical analyses were carried out in SPSS version 17.0 (SPSS, Inc., Chicago, IL).

Interaction between use of carboplatin and Ki- 67 was graphically evaluated by use of Subpopulation Treatment Effect Pattern Plot (STEPP) methodology [34]. Briefly, the STEPP method uses a sliding-windows approach to define several overlapping subpopulations of patients according to a continuous covariate, such as Ki-67, and plots the resulting treatment effects estimated within each subpopulation. The STEPP analyses were carried out with the R (http://cran.r-project.org/) software with Package ‘STEPP’.
